# Barley beta-glucan promotes MnSOD expression and enhances angiogenesis under oxidative microenvironment

**DOI:** 10.1111/jcmm.12442

**Published:** 2014-11-11

**Authors:** Silvia Agostini, Elena Chiavacci, Marco Matteucci, Michele Torelli, Letizia Pitto, Vincenzo Lionetti

**Affiliations:** a Laboratory of Medical Science, Institute of Life Sciences, Scuola Superiore Sant'AnnaPisa, Italy; b Institute of Clinical Physiology, National Council of ResearchPisa, Italy; c Division of Research & Development, Pastificio Attilio Mastromauro Granoro s.r.l.Corato, Italy; d Fondazione Toscana “G. Monasterio”Pisa, Italy

**Keywords:** beta-glucan, angiogenesis, endothelial cells, antioxidants, histone deacetylases

## Abstract

Manganese superoxide dismutase (MnSOD), a foremost antioxidant enzyme, plays a key role in angiogenesis. Barley-derived (1.3) β-d-glucan (β-d-glucan) is a natural water-soluble polysaccharide with antioxidant properties. To explore the effects of β-d-glucan on MnSOD-related angiogenesis under oxidative stress, we tested epigenetic mechanisms underlying modulation of MnSOD level in human umbilical vein endothelial cells (HUVECs) and angiogenesis *in vitro* and *in vivo*. Long-term treatment of HUVECs with 3% w/v β-d-glucan significantly increased the level of MnSOD by 200% ± 2% compared to control and by 50% ± 4% compared to untreated H_2_O_2_-stressed cells. β-d-glucan-treated HUVECs displayed greater angiogenic ability. *In vivo*, 24 hrs-treatment with 3% w/v β-d-glucan rescued vasculogenesis in *Tg* (*kdrl: EGFP) s843Tg* zebrafish embryos exposed to oxidative microenvironment. HUVECs overexpressing MnSOD demonstrated an increased activity of endothelial nitric oxide synthase (eNOS), reduced load of superoxide anion (O_2_^−^) and an increased survival under oxidative stress. In addition, β-d-glucan prevented the rise of hypoxia inducible factor (HIF)1-α under oxidative stress. The level of histone H4 acetylation was significantly increased by β-d-glucan. Increasing histone acetylation by sodium butyrate, an inhibitor of class I histone deacetylases (HDACs I), did not activate MnSOD-related angiogenesis and did not impair β-d-glucan effects. In conclusion, 3% w/v β-d-glucan activates endothelial expression of MnSOD independent of histone acetylation level, thereby leading to adequate removal of O_2_^−^, cell survival and angiogenic response to oxidative stress. The identification of dietary β-d-glucan as activator of MnSOD-related angiogenesis might lead to the development of nutritional approaches for the prevention of ischemic remodelling and heart failure.

## Introduction

In humans, the chronic exposure to reactive oxygen species (ROS) is a major risk factor for the impairment of endothelial function [1] and angiogenesis [2], which are both essential in self-repair ability of ischemic heart [3]. Even if ROS activates angiogenesis [2], compelling evidence indicates that the endothelial dysfunction because of superoxide anion (O_2_^−^) overload represents a mechanism for deregulated capillary formation after ischemia [4]. The safe and effective modulation of ROS-driven angiogenesis is still a challenging achievement, despite significant advances in the past two decades. High levels of the manganese superoxide dismutase (MnSOD), a major antioxidant enzyme, enhance the endothelial resistance to oxidative stress [5,6] and angiogenesis *via* the scavenging mitochondrial O_2_^−^ [7,8]. Thus, MnSOD is a promising candidate target for modulation of ROS-induced angiogenesis. To date, cellular MnSOD levels are significantly increased by a gene therapy approach [9,10] or *via* treatment with histone deacetylases (HDAC) inhibitors leading to cardioprotection through histone acetylation [11], which allows the expression of anti-apoptotic and angiogenic paracrine factors *in vitro* and *in vivo* [12]. However, a natural activator of MnSOD-related angiogenesis under oxidative stress is more desirable.

Barley-derived (1.3) beta-d-glucan (β-d-glucan), a natural water-soluble chain of d-glucose monomers linked by β-glycosidic bonds [13], lowers either oxidative stress [14] or serum cholesterol level in a dose-dependent manner [15]. The daily intake of β-d-glucan (1.5–4%) similarly induces the above-mentioned benefits in animal models and humans [16,17]. To date, it is unknown whether β-d-glucan promotes the MnSOD expression and the angiogenic ability of mature endothelial cells. Despite some controversial preliminary evidence [18], it has been suggested that long-term treatment with β-d-glucan enhances MnSOD-related angiogenesis under oxidative stress. For this purpose, we used the combination of two gold standard angiogenic tests, such as the *in vitro* human umbilical vein endothelial cells (HUVECs) matrigel assay [12,19,20] and the *in vivo* zebrafish angiogenesis assay [21,22].

## Materials and methods

### Chemicals

Barley-derived (1.3) β-d-glucan, hydrogen peroxide (H_2_O_2_) and sodium butyrate (NaBu) were purchased from Sigma-Aldrich Chemical Co (MO, USA).

### Endothelial cell cultures

Human umbilical vein endothelial cells (Cambrex Bio Science Inc, Walkersville, MD, USA) and human cardiac microvascular endothelial cells (HMVEC-C, Lonza, Milan, Italy) were cultured in endothelial growth medium (EGM-2 medium from Lonza) at 37°C in a humidified atmosphere of 5% CO_2_. Cells were exposed to the culture medium with 10% foetal bovine serum for at least 1 day before experiments. All assays were conducted using low cell passage cells (2–5 passages).

### Cell viability assay

Low-dose treatment with H_2_O_2_ (50 μM) was used to induce chronic oxidative stress on HUVECs [23,24]. After 24 hrs of exposure to H_2_O_2_ with or without 3% w/v β-d-glucan, cell viability was assessed by the Trypan blue dye exclusion test, as previously described [25]. When indicated, H_2_O_2_-stressed HUVECs were cultured in the presence of NaBu, a specific inhibitor of class I HDAC (reviewed in [26]), at rising concentrations (5–500 μM). All measurements were performed in triplicate.

### Dihydroethidium staining

Endothelial superoxide anion generation was determined by staining of HUVECs with fluorescent-labelled dihydroethidium (DHE; Invitrogen, CA, USA), according to manufacturer's instructions.

### Superoxide anion assay

Superoxide anion levels were assessed using a Superoxide Anion Assay Kit (Sigma-Aldrich Chemical Co), according to manufacturer's instruction relative to testing changes in superoxide anion production directly on intact cells.

### Western blotting

Equal amounts of protein extracted with RIPA buffer from cell pellets were processed for western blotting assay. The ratio of phospho-Ser1177eNOS (p-eNOS) and total eNOS, a hallmark of eNOS activity [27], was then determined as previously described [28]. Protein bands on immunoblots were quantified using ImageJ software.

### Nitric oxide detection

DAF-FM staining for the determination of intracellular nitric oxide bioavailability in human endothelial cells was performed as described elsewhere [29].

### *In vitro* angiogenesis assay

The *in vitro* angiogenesis assay was performed as described previously [12,19,20]. Analysis of capillary-like tube formation was performed using gel-precoated wells (Cultrex® Basement Membrane Extract, BME, Thema). Image analysis was performed by ImageJ software.

### Zebrafish lines, imaging and stages

Zebrafish transgenic embryos Tg (kdrl:EGFP) s843Tg (expressing EGFP in the vascular system) were raised and maintained under standard laboratory conditions [30] as described elsewhere [31]. The oxidative stress was induced *in vivo* by treatment with PMA, an agonist of protein kinase C [32]. Imaging was performed on zebrafish embryos at 24 hpf (hours post-fertilization). Investigation was approved by the Animal Care Committee of the Italian Ministry of Health in accordance with the European law (EU 63-2010) and with the *Guide for the Care and Use of Laboratory Animals* published by the US National Institutes of Health (NIH Publication No. 85–23, revised 1996).

### Statistical analysis

The statistical analysis was performed using GraphPad Prism ver. 5. All results are presented as mean ± SD. Statistical comparisons were made by anova and Dunnett's Multiple Comparison Test was used as the post-hoc test. *P* < 0.05 was considered statistically significant.

## Results

### β-d-glucan treatment promotes endothelial cell survival and MnSOD expression under chronic oxidative stress

**Fig. 1:**
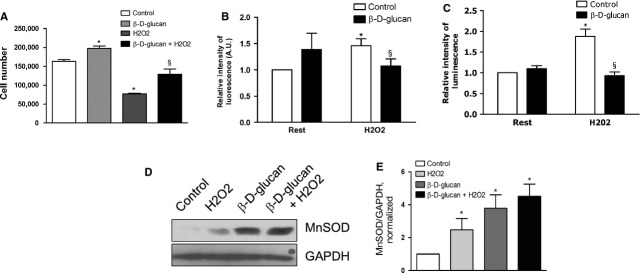
β-d-glucan treatment promotes endothelial cell survival and MnSOD expression under chronic oxidative stress. (**A**) HUVECs survival after 24 hrs treatment with 50 μM H_2_O_2_, alone or in combination with 3% β-d-glucan. Untreated and unstressed cells were used as control. (**B**) Quantification of the relative intensity of fluorescence in DHE-positive cells, compared to control condition. (**C**) Quantification of the relative intensity of luminescence in cells producing superoxide anion, compared with control condition. (**D**) Representative western blot bands for MnSOD and GAPDH. (**E**) Measurement of the level of MnSOD expression normalized over loading control (GAPDH). (mean ± SD; *n* = 4) *P < 0.05 *versus* control; §*P* < 0.05 *versus* H_2_O_2_.

As shown in Figure 1A, the treatment with 3% β-d-glucan significantly increased the number of viable cells under oxidative stress.

The measure of fluorescence in the nuclei of cells positive for dEDT staining showed that β-d-glucan normalized the ROS load of stressed cells (Fig. 1B) while such amount was significantly increased in the absence of treatment. In addition, we confirmed that β-d-glucan reduced the total O_2_^−^ in HUVECs (Fig. 1C) and HMVEC-C (Fig. S1). β-d-glucan significantly enhanced the protein expression of MnSOD in viable cells exposed to normal microenvironment. The level of MnSOD in β-d-glucan-treated cells exposed to H_2_O_2_ was tendentially higher compared to untreated stressed cells (Fig. 1C, quantified in 1D).

### β-d-glucan treatment increases eNOS phosphorylation and histone H4 acetylation in viable endothelial cells

**Fig. 2:**
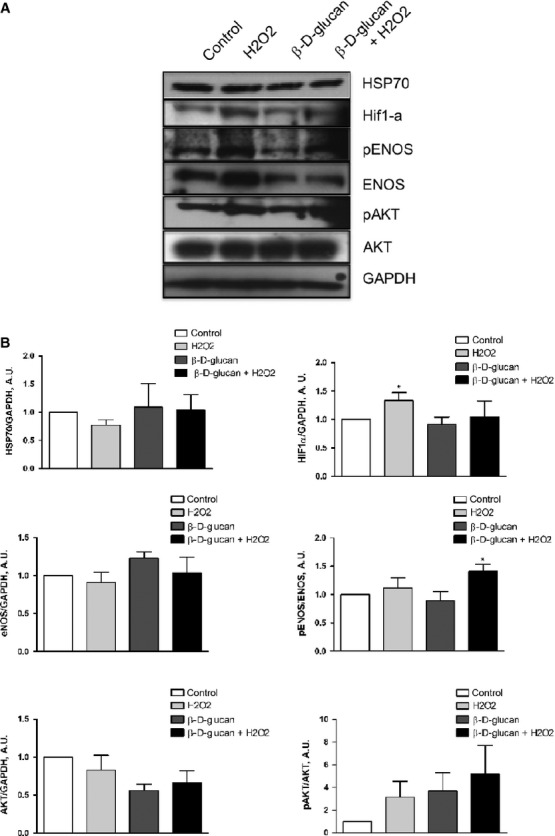
β-d-glucan treatment increases eNOS phosphorylation and histone H4 acetylation in viable endothelial cells. (**A**) Representative western blot bands for HSP70, HIF1-α, p-AKT, AKT, p-eNOS, eNOS and GAPDH in each experimental condition. (**B**) Measurement of the level of HSP70, HIF1-α, AKT, eNOS expression normalized over loading control (GAPDH). Phosphorylation level of eNOS and AKT was quantified normalizing the amount of phosphorylated protein over total protein: p-eNOS/eNOS and p-AKT/AKT. (mean ± SD; *n* = 4) *P < 0.05 *versus* control.

**Fig. 3:**
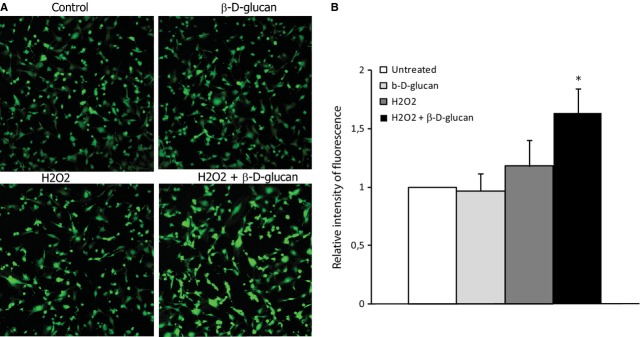
β-d-glucan treatment increases nitric oxide generation in stressed endothelial cells. (**A**) Representative images of DAF staining of HUVECs at rest or with H_2_O_2_, in the presence of β-d-glucan or vehicle. (**B**) Quantification of the relative intensity of fluorescence in DAF-FM diacetate positive cells at rest or during oxidative stress (H_2_O_2_), in the presence of β-d-glucan or vehicle. (mean ± SD; *n* = 3) *P < 0.05 *versus* control.

**Fig. 4:**
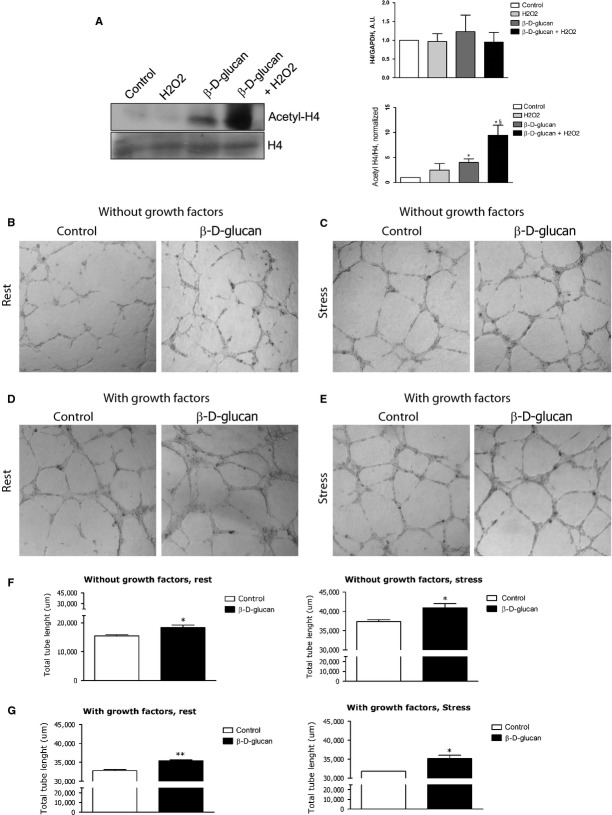
β-d-glucan treatment increases histone H4 acetylation and promotes human capillary formation *in vitro*. (**A**) Representative western blot bands for histone H4 (H4) and acetylated histone H4 (Acetyl-H4) in each experimental condition. Measurement of the level of H4 expression normalized over loading control (GAPDH). Acetylation level of H4 was quantified normalizing the amount of Acetyl-H4 protein over total protein. (**B** and **C**) Representative images of tube formation from HUVECs without exogenous growth factors. (**D** and **E**) Representative images of tube formation from HUVECs with exogenous growth factors. (**F**) Measure of total length of tubes from HUVECs without exogenous growth factors. (**G**) Measure of total length of tubes from HUVECs with exogenous growth factors. Intrinsic tube formation ability was tested alone (control) or with 3% β-d-glucan; at rest (left side) or during oxidative stress (stress, right side) (mean ± SD; *n* = 4) *P < 0.05 *versus* control; **P < 0.01 *versus* control; §*P* < 0.05 *versus* H_2_O_2_.

β-d-glucan treatment of stressed cells caused a significant increase in p-eNOS/eNOS ratio without affecting the level of p-AKT, AKT, and heat shock protein-70 (HSP-70; Fig. 2A, quantified in 2B). We detected a significant increase in nitric oxide production in stressed β-d-glucan-treated HUVECs (Fig. 3) and HMVEC-C (Fig. S1). Interestingly, the rise of hypoxia inducible factor (HIF)-1α level was prevented in H_2_O_2_-stressed cells treated with β-d-glucan (representative blots shown in Fig. 2A, quantified in 2B). As shown in Figure 4A, the level of H4 acetylation in β-d-glucan-treated cells was significantly higher than untreated cells under normal and oxidative microenvironment.

### β-d-glucan treatment promotes human capillary formation *in vitro*

β-d-glucan increased the tube formation activity of normal endothelial cells cultured without exogenous endothelial growth factors (Fig. 4B). As shown in Figure 4B (right panel), β-d-glucan induced a significant slight increase in ROS-driven angiogenesis.

In the presence of exogenous endothelial growth factors (Fig. 4C), the formation of capillaries from normal or H_2_O_2_-stressed HUVECs was significantly increased by treatment with 3% β-d-glucan. The addition of exogenous growth factors in the culture medium did not increase the magnitude of ROS-driven angiogenesis (Fig. 4C, right panel) nor enhances the pro-angiogenic effect of β-d-glucan. In addition, the treatment with a similar dose of β-d-glucan significantly increased the angiogenic ability of HMVEC-C *in vitro* (Fig. S1).

### β-d-glucan treatment rescues the vasculogenic activity under oxidative stress *in vivo*

**Fig. 5:**
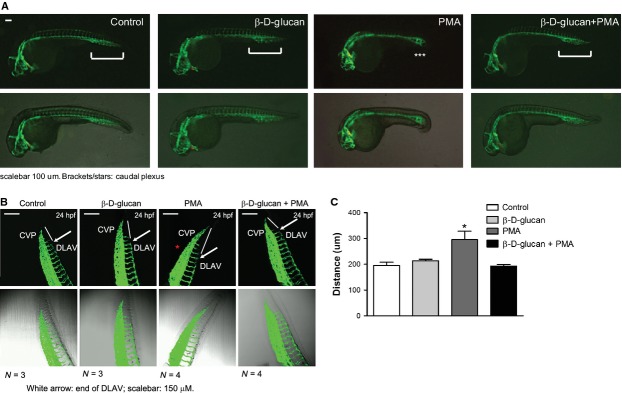
β-d-glucan treatment rescues the vasculogenic activity under chronic oxidative stress *in vivo*. (**A**) Representative images of Zebrafish transgenic Tg (kdrl: EGFP)^s843Tg^ embryos at 24 hpf (hours post-fertilization), alone (control) or treated at 70% epiboly stage with 3% β-d-glucan, PMA, and PMA+3% β-d-glucan. Brackets: normal caudal plexus; stars: injured caudal plexus; scale bar 100 μm. (**B**) At each experimental condition, representative images of caudal view of embryos, at 24 hpf. DLAV: dorsal longitudinal anastomotic vessel (CVP, caudal vein plexus). White arrow: end of DLAV; scale bar 150 μm. (**C**) Measurement of the distance between the end of DLAV and the tip of CPV at each experimental condition. *P < 0.05 *versus* control.

The chronic exposure to PMA alone significantly halted the formation of the caudal artery at 24 hpf, measured as an increase in the distance between the end of the dorsal longitudinal anastomotic vessel (DLAV) and the caudal vein plexus (CVP) of Tg (*kdrl: EGFP*)^s843Tg^ embryos (Fig. 5). In an additional group, 3% β-d-glucan counteracted the ROS-induced vascular toxicity and preserved the vasculogenic activity essential to lead the normal formation of the caudal artery (Fig. 5A, quantified in Fig. 5B). No impairment of vasculogenesis was detected in samples from β-d-glucan -treated embryos under normal conditions.

### β-d-glucan modulation of cell viability and MnSOD expression does not rely on histone acetylation

**Fig. 6:**
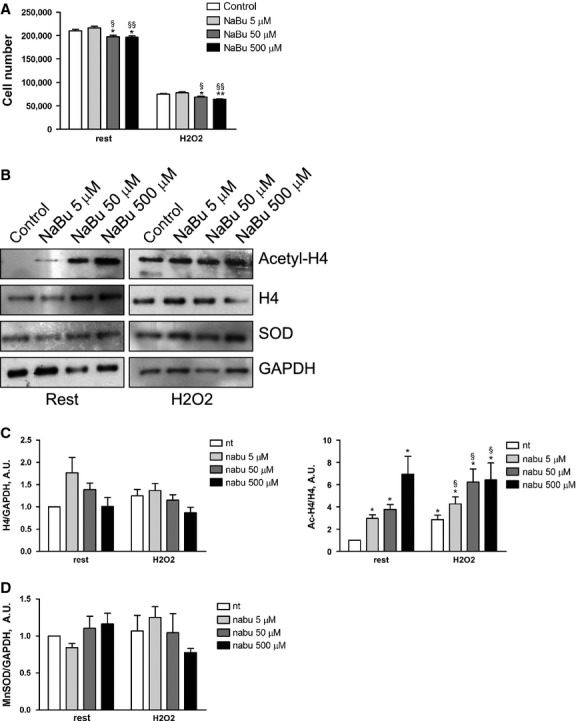
NaBu does not increase Endothelial MnSOD expression. (**A**) HUVECs survival after 24 hrs treatment with 50 μM H_2_O_2_, alone or with increasing dose of sodium butyrate (NaBu) ranging from 5 to 500 μM. (**B**) Representative western blot bands for MnSOD, histone H4 (H4), acetylated histone H4 (Ac-H4) and GAPDH in each experimental condition. (**C**) Measurement of the level of H4 expression normalized over loading control (GAPDH). Acetylation level of H4 was quantified normalizing the amount of Acetyl-H4 protein over total protein. (**D**) Measurement of the level of MnSOD expression normalized over loading control (GAPDH). nt: control (mean ± SD; *n* = 4) *P < 0.05 *versus* control; **P < 0.01 *versus* control: ***P < 0.001 *versus* control; §*P* < 0.05 *versus* H_2_O_2_.

In both microenvironmental conditions, 24 hrs treatment with 50 and 500 μM of NaBu had a slight but significant adverse effect on cell survival (Fig. 6A); although, 5 μM of NaBu did not affect the cell viability. As shown in Figure 6B and quantified in Figure 6C, NaBu increased the level of H4 acetylation in a dose-dependent manner without changing the endothelial MnSOD expression level in all the experimental conditions.

**Fig. 7:**
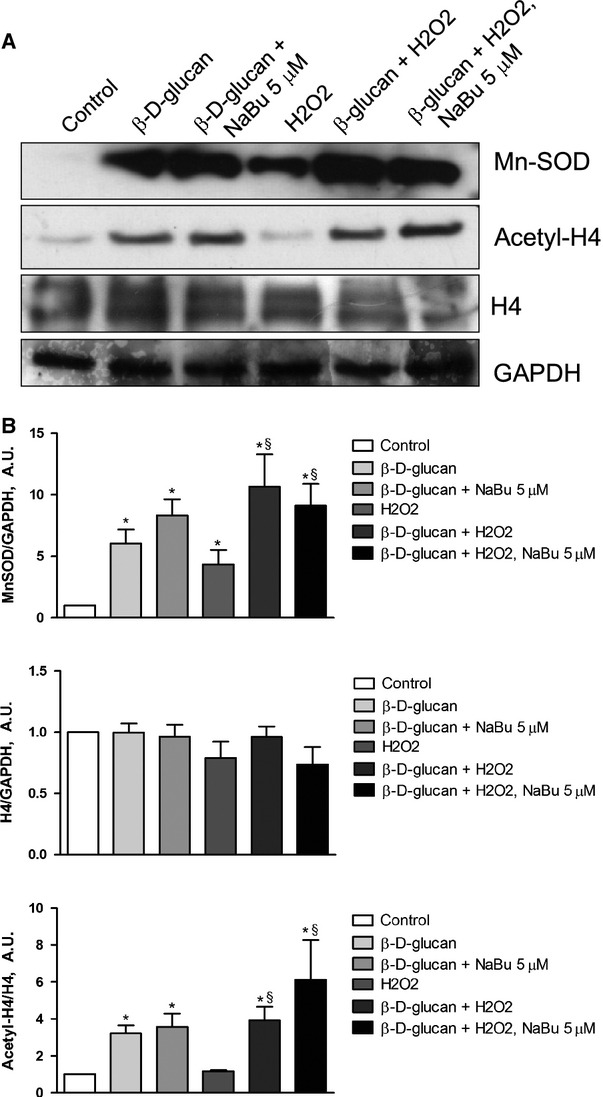
NaBu does not affect β-d-glucan-induced MnSOD up-regulation under chronic oxidative stress. (**A**) Representative western blot bands for MnSOD, histone H4 (H4), acetylated histone H4 (Ac-H4) and GAPDH in each experimental condition. (**B**) Measurement of the level of MnSOD and H4 expression normalized over loading control (GAPDH). Acetylation level of H4 was quantified normalizing the amount of Acetyl-H4 protein over total protein. Cells were exposed to H_2_O_2_ for 24 hrs, alone or in combination with 3% β-d-glucan, NaBu (5 μM), or both. Unstressed and untreated cells were used as control (mean ± SD; *n* = 4). *P < 0.05 *versus* control; §*P* < 0.05 *versus* H_2_O_2_.

**Fig. 8:**
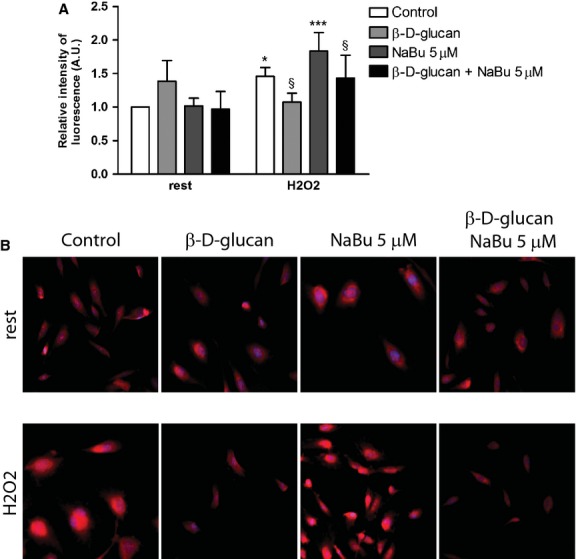
NaBu does not affect β-d-glucan-induced superoxide anion down-regulation under chronic oxidative stress. (**A**) Quantification of the relative intensity of fluorescence in DHE positive cells in each experimental condition at rest or during oxidative stress (H_2_O_2_). (**B**) Representative images of DHE staining of HUVECs at rest or with H_2_O_2_, in the presence of either β-d-glucan, NaBu (5 μM), or both (mean ± SD; *n* = 4). *P < 0.05 *versus* control; ***P < 0.001 *versus* control; §*P* < 0.05 *versus* H_2_O_2_.

The simultaneous 24 hrs treatment of HUVECs with 3% w/v β-d-glucan and NaBu 5 μM did not interfere with β-d-glucan effects on cell viability (data not shown) and MnSOD expression (Fig. 7A, quantified in Fig. 7B, upper panel) even in the presence of high level of H4 acetylation (Fig. 7A, quantified in Fig. 7B, lower panel). Finally, the treatment of cells with 3% w/v β-d-glucan, alone or in combination with NaBu 5 μM, prevented the rise of O_2_^−^ level (Fig. 8A for quantification of dEDT staining; Fig. 8B for representative images).

### β-d-glucan modulation of human capillary formation does not rely on histone acetylation

**Fig. 9:**
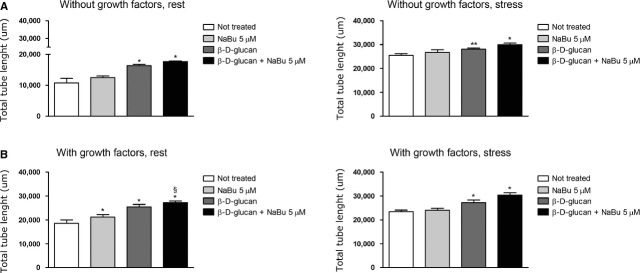
NaBu does not affect β-d-glucan-induced capillary formation under chronic oxidative stress. (**A**) *In vitro* angiogenesis assay without exogenous growth factors. Intrinsic tube formation ability was tested, alone (untreated) or with 3% β-d-glucan, NaBu (5 μM,) or both; at rest (left side) or during oxidative stress (right side). (**C**) *In vitro* angiogenesis assay with exogenous growth factors. Tube formation ability was tested, alone (untreated) or in combination with 3% β-d-glucan, NaBu (5 μM), or both; at rest (left side) or during oxidative stress (right side; mean ± SD; *n* = 4). *P < 0.05 *versus* control; §*P* < 0.05 *versus* β-d-glucan.

Even if the 24 hrs treatment with NaBu 5 μM did not attenuate the angiogenic ability of HUVECs, it slightly increased capillary formation from HUVECs cultured without growth factors and stress (Fig. 9A). We found that the inhibition of HDAC activity did not affect the proangiogenic effect of β-d-glucan. As shown in Figure 9B, the co-treatment of cells with β-d-glucan and NaBu 5 μM increased the capillary forming activity of normal HUVECs cultured in complete medium.

## Discussion

The enhancement of endogenous angiogenic activity may hold promises for restoring adequate myocardial perfusion in ischemic heart. It is emerging that the optimization of antioxidant gene expression may promote mature capillary formation [33]. MnSOD is the most effective antioxidant enzyme [34] that protects myocardium *via* scavenging mitochondrial O_2_^−^ [35]. Cardiac overexpression of MnSOD limits cardiac cell loss and renders the heart more resistant to the oxidative burst [36]. In addition, MnSOD gene transfer restores endothelial function through increasing activity of eNOS [37], which exerts direct pro-angiogenic effect [38]. Consistent with these observations, it has been suggested that increased MnSOD expression might enhance ROS-driven angiogenesis.

A recent study suggested that the use of natural molecules could be sufficient to induce endothelial MnSOD expression [39]. Hence, the identification of novel dietary compounds able either to increase MnSOD level or to enhance angiogenesis may prove rewarding in sight of therapeutic myocardial angiogenesis, therefore, avoiding invasive approaches.

We provided evidence that barley β-d-glucan is an active natural enhancer of the angiogenic potential of ROS-exposed endothelial cells related to increased MnSOD expression.

The treatment of monolayered H_2_O_2_-stressed HUVECs with 3% w/v β-d-glucan increased MnSOD level without reducing the expression of HSP70, a stress protein sufficient to active MnSOD [40]. In agreement with previous studies, the increase in the expression of MnSOD was useful to improve its enzymatic activity [41], which prevails over that of SOD1 in attenuating superoxide anion level [42]. The rise of β-d-glucan-induced MnSOD expression, which reduced the intracellular load of O_2_^−^ in stressed endothelial cells, was related to the normal level of HIF-1α, thus rendering the cells more resistant to the oxidative burst. In fact, the reduction in O_2_^−^ level suppressed the expression of HIF-1α [43], which plays a role in promoting cell death by autophagy [44]. On the other hand, the eNOS activation *via* phosphorylation of serine 1177 was enhanced by β-d-glucan without involving the activation of AKT/PKB, which is an established eNOS activator [27,45]. Increased nitric oxide generation contributes to neutralize O_2_^−^ in stressed cells [46], and the endothelial eNOS activation is also regulated in an AKT-independent manner [47]. In our study, the nitric oxide synthesis was significantly increased in stressed β-d-glucan- treated adult and newborn endothelial cells. Therefore, we have shown that β-d-glucan-induced MnSOD up-regulation encompasses both endothelial pro-survival features, by preventing the increase in HIF-1α expression, and the potential to reduce superoxide anion level, by activating eNOS in an AKT-independent manner.

Taking into account that the β-d-glucan effects were considerably more accentuated under oxidative stress than in normal conditions, we tested whether the increased MnSOD expression affects the angiogenic response to ROS. Notably, β-d-glucan-treated endothelial cells generated more vessels either with or without exogenous growth factors in the medium. In addition, we found that the extent of increasing tube formation from cultured HMVEC-C treated by similar dose of β-d-glucan was smaller than HUVECs (Fig. S1). Our data were in accordance with a previous study showing lower angiogenic response to exogenous factors of adult endothelial cells compared to HUVECs [48]. The finding that β-d-glucan-treated cells exhibited a significant yield of angiogenesis in the absence of either exogenous growth factors or oxidative stress prompted the hypothesis that β-d-glucan exerts a direct pro-angiogenic activity. It is known that endothelial cells form a dense capillary network regardless the conventional paracrine growth factors [19,49,50]. Since MnSOD overexpression promotes endothelial cell sprouting [41], it is conceivable that β-d-glucan-induced angiogenesis depends upon MnSOD level. In addition, we cannot exclude the direct pro-angiogenic effect also exerted by NO generated from eNOS [38], which is activated by MnSOD [37].

To confirm *in vitro* data, we performed *in vivo* experiments using transgenic zebrafish embryos. In an additional experiment, we treated PMA-exposed embryos with 3% w/v β-d-glucan. Prolonged PMA treatment inhibits VEGF expression [51] and VEGF-induced angiogenesis by down-regulation of PKC [52]. In our model, long-term PMA exposure remarkably hampered the development of the dorsal longitudinal anastomotic vessel and the simultaneous treatment of PMA-exposed embryos with β-d-glucan rescued the physiological vascular development. β-d-glucan did not affect vessel density of healthy embryos.

Unraveling mechanisms would offer options to better modulate MnSOD-related angiogenesis by β-d-glucan. Endothelial cells express dectin-1, a C-type lectin-like receptor, [53] and β-d-glucan effects are suppressed with Dectin-1-specific blocking monoclonal antibody [54]. It suggests that the engagement of Dectin-1 might represent a major initial step in endothelial gene expression modulation. It is worth noticing that β-d-glucan-treated HUVECs showed a consistent histone acetylation level, yet it was higher in H_2_O_2_-exposed cells. Our results prompted the hypothesis that the binding of Dectin-1 to β-d-glucan induces MnSOD expression *via* histone acetylation. Shimazu *et al*. [55], in fact, have shown that histone acetylation as a result of inhibition of class I HDAC increases MnSOD expression. In our model, with rising of histone acetylation level, the MnSOD expression was unaffected by increasing doses of NaBu, a class I HDAC inhibitor. At higher doses of NaBu (50 or 500 μM), we detected cell death in the presence of higher level of histone acetylation and O_2_^−^ (data not shown). Conversely, the exposure of HUVECs to a lower dose of NaBu (5 μM) added to complete medium enhanced angiogenesis. Nonetheless, the β-d-glucan effects were unaffected by co-treatment with 5 μM NaBu.

Our study verified that the β-d-glucan-induced rise of MnSOD level and tube formation was not because of inhibition of class I HDAC. Even if the pro-angiogenic effect of water-soluble β-d-glucan was independent of HDAC activity, we cannot exclude that higher doses of β-d-glucan may promote cell death similarly to NaBu. In fact, a previous study demonstrated that treatment of HUVECs with a cocktail containing a higher dose of fungal β-d-glucan exhibited an anti-angiogenic effect [18].

Studies are in progress to further dissect the panel of signaling pathways modulating gene profile recruited by barley β-d-glucan.

In conclusion, we identified barley β-d-glucan as natural activator of MnSOD expression and the angiogenic ability of ROS-exposed endothelial cells regardless histone acetylation. Since mature endothelial cells exhibit intrinsically lower expression of MnSOD compared to endothelial progenitor cells [56], our results assume significance to develop β-d-glucan-based approaches of therapeutic angiogenesis for the prevention of heart failure.

## Supporting information

**Figure S1** Effects of β-d-glucan treatment on cardiac HMVECs.
