# Supplementary material for: Barley beta-glucan promotes MnSOD expression and enhances angiogenesis under oxidative microenvironment
Source: J Cell Mol Med. 2014 Nov 11;19(1):227–38. doi: 10.1111/jcmm.12442 (PMC4288365; doi:10.1111/jcmm.12442)
Supplement: Supplementary file 1 — Figure S1 Effects of β-d-glucan treatment on cardiac HMVECs. [file jcmm0019-0227-sd1.docx]

**SUPPORTING INFORMATION**

**Chemicals**

Sodium butyrate was dissolved in distilled water at 50 mM concentration as a stock solution and stored at -20°C. Phorbol-12-myristate-13-acetate (PMA, Sigma Chemical Co, MO, USA) was dissolved in dimethyl-sulfoxide (DMSO) at a concentration of 62 mg/ml and stored at -20°C until needed for experiments.

**Western Blotting**

Before performing western blot analyses, cell pellets were lysed in RIPA 150 mM NaCl buffer containing protease inhibitors (Pierce). Total protein concentration was assessed using BCA Protein Assay Kit (Pierce). Equal amounts of protein (30 μg) were resolved on 12 % SDS-polyacrylamide gel and transferred to polyvinylidene difluoride (PVDF) membrane. Membranes were blocked with 5 blocking reagent in TBS/Tween (0,01%) at room temperature for 1 hour, and then incubated with primary antibodies at a predetermined concentration overnight at 4 C°. Primary antibodies were used to detect phospho-AKT (1:2000, Cell Signaling, Danvers, MA), total AKT (1:1000, Cell Signaling, Danvers, MA), total endothelial nitric oxide synthase (eNOS, 1:1000, BD biosciences, Franklin Lakes, NJ), phospho-Ser1177-eNOS (1:1000, Cell Signaling, Danvers, MA), hypoxic inducible factor 1-alpha (HIF1-α, 1:500, Santa Cruz Biotechnology, Inc, USA), Manganese Superoxide Dismutase (MnSOD, 1:1000; Millipore, MA, USA), total histone type H4 (1:1000, AbCam Inc., Cambridge, UK), pan-acetylated histone type H4 (Millipore, MA, USA) and alpha-tubulin (1:1000, Santa Cruz Biotechnology, Inc, USA). All incubations were performed in the proper blocking (non-fat skim milk 5%, Euroclone S.p.A., Italy), or BSA 3% (Sigma Aldrich Chemical Co (MO, USA)) reagent as indicated by primary antibody manufactor. Secondary antibodies were incubated at the appropriate concentrations for 1 hour at room temperature. Horseradish peroxidase-conjugated anti-rabbit or anti-mouse secondary antibodies were from Abcam Inc., Cambrige, UK. Specific protein bands were detected using ECL Plus Western blotting Detection System (Bio-Rad Laboratories, Inc., CA, USA).

**Dihydroethidium Staining**

Positive nuclear DHE staining is an indicator of superoxide generation in cells. 5 images of cells were captured randomly by fluorescent microscope (Olympus BX) with a setting of 40× magnification. The nuclear fluorescence in DHE positive cells was quantified.

Superoxide Anion Assay

Human endothelial cells were seeded in T75 flasks and mantained in EGM2 medium. At 70% confluence, cells were treated with H2O2 50 μM and/or 3% β-D-glucan, and incubated for 24 hours. Thereafter, cells were trypsinized, counted and 5x105 cells were processed for superoxide anion measurement. Luminescence emission was measured using a GloMax Luminometer (Promega) using the standard luminescence protocol. The experiments were performed in triplicate.

**Nitric Oxide detection**

Human endothelial cells were plated on glass-bottomed will-co plates (Willco Wells). For oxidative stress induction, cells were treated with 50 μM H2O2 for 24 hours, either alone or in presence of 3% β-D-glucan. After incubation, cells were loaded with 10 μM of the NO-sensitive fluorescence probe DAF-FM (Sigma) at 37°C for 30 min. The medium, containing loading dye, was removed and replaced with EGM-2 medium and incubated for another 30min at 37°C to ensure complete de-esterification. Fluorescence was detected within 10 fields for each condition; relative fluorescence intensity per cell was quantified using ImageJ software. The experiments were performed in triplicate.

**In vitro angiogenesis assay**

To evaluate the effect of β-D-glucan on angiogenic capacity of hydrogen peroxide-stimulated HUVECs, 3% w/v β-D-glucan was added to the cultured cells for 24 hours in the presence of H2O2. In control conditions, H2O2-stimulated HUVECs were cultured in the presence of EGM-2. Capillary-like structures were observed over the following 24-hour period and were photographed using a light microscope equipped with a digital sight camera (Nikon).

**Zebrafish lines, imaging and stages**

Embryos 70% epiboly stage were dechorionated and treated ON at with w/v β-D-glucan or PMA 200 ng/ml or 3% w/v β-D-glucan + PMA 200 ng/ml diluted in zebrafish maintenance water. Embryos receiving DMSO (0.2%) served as vehicle controls and were equivalent to no treatment. Each experiment was repeated at least three times, with 10 embryos per group. Embryos were maintained using standard methods.

Imaging were performed on zebrafish embryos at 24hpf (hours post fertilization) as follow: embryos were embedded in low melting agar 1% (sigma) and images were acquired with a Leica DM IRE 2 confocal microscope, image stacks were processed with ImageJ by projection.

FIGURE LEGEND

**Supporting Figure 1. Effects of β-D-glucan treatment on cardiac HMVECs.**

A: quantification of the relative intensity of luminescence in control and β-D-glucan- treated cells producing superoxide anion, at rest or after 24 hours treatment with 50 μM H2O2. B: quantification of the relative intensity of fluorescence in DAF-FM diacetate stained cells at rest or 24 hours treatment with 50 μM H2O2. Where indicated, cells were treated with 3% b-D-glucan. C: measure of total length of tubes from HMVECs without exogenous growth factors. D: measure of total length of tubes from HMVECs with exogenous growth factors. Intrinsic tube formation ability was tested alone (control) or with 3% β-D-glucan; at rest (left side) or during oxidative stress (stress, right side). (mean±S.D.; n=4) *: p<0.05 vs control; ***: p<0.001 vs control.
